# Interictal spikes and evoked cortical potentials share common spatiotemporal constraints in human epilepsy

**DOI:** 10.3389/fnetp.2025.1602124

**Published:** 2025-05-30

**Authors:** Samuel B. Tomlinson, Patrick Davis, Caren Armstrong, Michael E. Baumgartner, Benjamin C. Kennedy, Eric D. Marsh

**Affiliations:** ^1^ Department of Neurosurgery, Perelman School of Medicine, University of Pennsylvania, Philadelphia, PA, United States; ^2^ Division of Neurology, Children’s Hospital of Philadelphia, Philadelphia, PA, United States; ^3^ Department of Neurology, University of California, Davis, Sacramento, CA, United States; ^4^ Perelman School of Medicine, University of Pennsylvania, Philadelphia, PA, United States; ^5^ Division of Neurosurgery, Children’s Hospital of Philadelphia, Philadelphia, PA, United States; ^6^ Departments of Neurology and Pediatrics, Perelman School of Medicine, University of Pennsylvania, Philadelphia, PA, United States

**Keywords:** interictal epileptiform discharge, epilepsy, seizure, network, stimulation, cortico-cortical evoked potentials

## Abstract

Interictal epileptiform discharges (IEDs) are pathologic hallmarks of epilepsy which frequently arise and spread through networks of functionally-connected brain regions. Recent studies demonstrate that the sequential recruitment of brain regions by propagating IEDs is highly conserved across repeated discharges, suggesting that IED propagation is spatiotemporally constrained by features of the underlying epileptic network. Understanding how repetitive IED sequences relate to the spatiotemporal organization of the epileptic network may reveal key insights into the pathophysiological role of IEDs during epileptogenesis. Delivery of exogenous electrical current allows for direct experimental probing of epileptic network circuitry and correlation with spontaneous epileptiform activity (e.g., IEDs). In this pilot study of human subjects with refractory epilepsy, we performed cortical stimulation via invasive depth electrodes to test whether spatiotemporal patterns observed during spontaneous IEDs are reproduced by evoked cortical potentials. We found that evoked potentials were accentuated following stimulation of early-activating “upstream” IED regions (anterograde) and attenuated with stimulation of late-activating “downstream” IED regions (retrograde). Concordance between IED latencies and evoked potentials suggests that these distinct network phenomena share common spatiotemporal constraints in the human epileptic brain.

## Graphical Abstract

**Figure:**
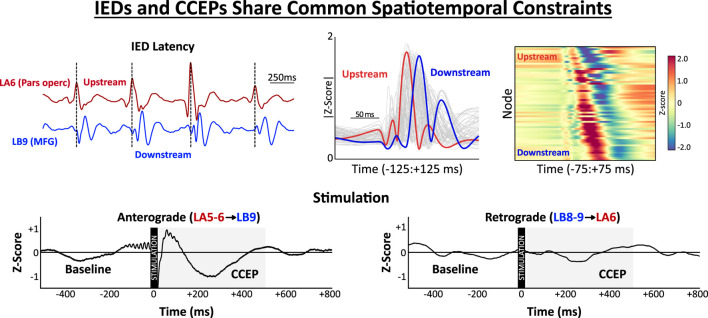

## 1 Introduction

The epileptic brain is increasingly conceptualized as a network defined by complex, dynamic patterns of connectivity distributed across both local (i.e., regional micro-circuits) and global (i.e., whole-brain connectomics) scales (Kramer and Cash, 2012). Analysis of human invasive stereoelectroencephalography (SEEG) recordings permits examination of epileptic network interactions spanning multiple distinct brain regions with high spatial and temporal resolution. Characterizing how epileptiform activity arises and propagates through the brain has tremendous potential for refining network models of epilepsy and revealing targets for surgical intervention.

Interictal epileptiform discharges (IEDs) are spontaneous paroxysms arising from the synchronous activation of hyper-excitable cortical ensembles in patients with epilepsy (de Curtis and Avanzini, 2001; Staley and Dudek, 2006). Simultaneous co-occurrence of IEDs between brain regions has been interpreted as evidence of functional connectivity, and the millisecond-scale latencies between IEDs may reflect asymmetric patterns of transmission across the IED-generating network (Diamond et al., 2019; Tomlinson et al., 2016). Previous studies demonstrate that IEDs propagate through repetitive spatiotemporal sequences that are stable across thousands of discharges over many hours of continuous recording (Tomlinson et al., 2019). Stereotyped patterns of spatiotemporal recruitment have been similarly demonstrated in other *in vivo* neural systems (e.g., rat auditory cortex (Luczak et al., 2009); primate visual cortex (Jermakowicz et al., 2009); human memory retrieval (Vaz et al., 2020), wherein the sequential activation of network nodes is highly conserved across repeated discharges. These findings support the view that the functional organization of neural networks, both in healthy and diseased states, can constrain the directional flow of spontaneous activity throughout the brain.

In the context of epilepsy, stereotyped patterns of sequential IED recruitment are postulated to reflect pathologic, plasticity-entrained circuits facilitating the directional transmission of IEDs from upstream activators (i.e., nodes exhibiting consistent early recruitment) towards downstream receivers (Diamond et al., 2024; Tomlinson et al., 2025). This polarized circuitry may be reinforced by repeated IEDs, which can occur hundreds or even thousands of times per day. Clinical studies examining seizure outcomes following resection consistently find that excision of upstream IED regions is associated with higher likelihood of seizure freedom, (Hufnagel et al., 2000; Alarcon et al., 1997; Matarrese et al., 2023; Shamas et al., 2023), supporting the intuitive notion that upstream and downstream IED regions differ in terms of intrinsic epileptogenicity. However, more precise experimental work is needed to characterize how IED recruitment latencies relate to the underlying spatiotemporal organization of the epileptic network.

Cortical stimulation provides an opportunity to directly probe interactions between brain regions by delivering exogenous current and recording cortico-cortical evoked potentials (CCEPs). In this manner, stimulation can be used to actively interrogate network circuitry inferred from the analysis of spontaneous epileptiform activity such as IEDs. Presently, little is known about the relationship between IED propagation and CCEPs. One hypothesis is that the epileptic network imposes common constraints on the directional flow of IEDs and CCEPs, such that spatiotemporal relationships observed during spontaneous IEDs are recapitulated by asymmetric responses to stimulation of upstream versus downstream nodes. An alternative hypothesis is that IEDs and evoked potentials are independent phenomena governed by distinct spatiotemporal mechanisms. To our knowledge, these opposing hypotheses have never been explicitly scrutinized in the human epileptic brain.

In this pilot study, we examined the relationship between spontaneous IED propagation and CCEPs in human subjects exhibiting continuous, rhythmic bursts of IEDs arising from distributed networks of co-activating gray matter structures. IED recruitment latencies were computed to distinguish upstream (early), intermediate, and downstream (late) IED nodes. Direct cortical stimulation was performed using repeated low-frequency (1 Hz) pulses delivered to adjacent SEEG contacts. In line with a common-constraints view of epileptic network polarization, we predicted that stimulation of upstream IED nodes would elicit broad network responses (“anterograde”) which would not be reciprocated by stimulation of intermediate and downstream nodes (“retrograde”).

## 2 Materials and methods

### 2.1 Human subjects

**FIGURE 1:**
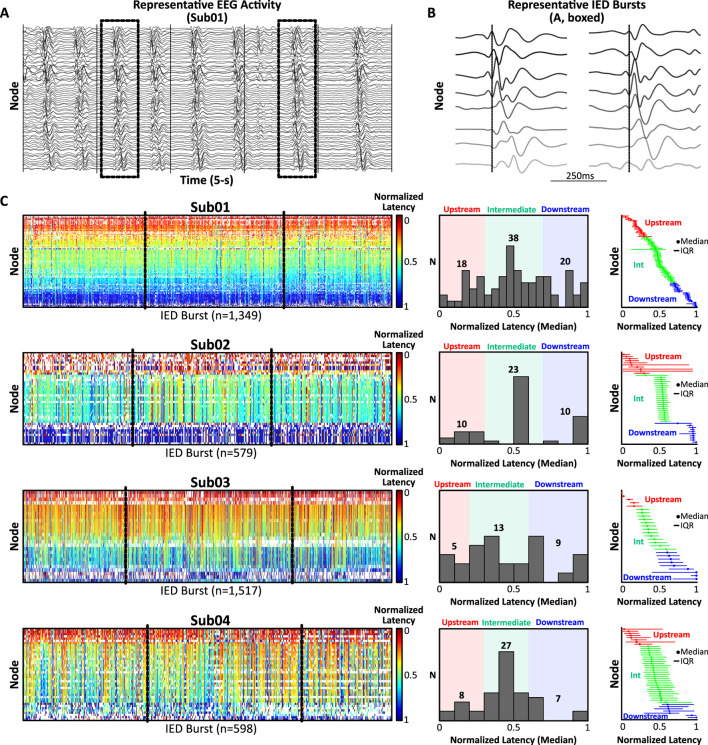
Spatiotemporal analysis of spontaneous IED bursts. **(A)** Representative SEEG activity (5-s, 1–50 Hz band-pass filter for visualization) from Sub01 exhibiting continuous, rhythmic bursts of diffusely-synchronous IEDs arising from a consistent network of co-activating nodes (n = 76). **(B)** Two representative IED bursts (boxed, A; 500 ms window) sorted by latency from discharge onset (dotted line) illustrating millisecond-scale lags in recruitment latencies across eight representative nodes. **(C)** Left: Normalized recruitment latency (0 = earliest IED in burst, 1 = terminal IED in burst) was computed for each node across all IED bursts (warmer colors = upstream, cooler colors = downstream). Middle: Histograms of median normalized recruitment latencies were used to classify nodes as upstream (red), intermediate (green), or downstream (blue). Right: Nodes rank-ordered by ascending median IED recruitment latency and classified as upstream, intermediate, or downstream. Abbreviations: IED, interictal epileptiform discharge; Int, intermediate; IQR, interquartile range.

To investigate the possibility that IEDs and CCEPs share common spatiotemporal constraints, we analyzed spontaneous IED propagation and performed systematic low-frequency (1 Hz) cortical stimulation in a unique pilot cohort of four subjects with medically-refractory epilepsy (2 females, 2 males, ages 3–10 years; Supplementary Table S1). The subjects selected for this pilot analysis came from a larger database of 51 pediatric patients who had undergone systematic low-frequency cortical stimulation on a research protocol approved by the Children’s Hospital of Philadelphia (CHOP) Institutional Review Board. Each pilot subject exhibited synchronous, continuous, rhythmic (0.5–2 Hz) bursts of IEDs propagating through a network of co-activating gray matter nodes via a single consistent spatiotemporal pattern, indicative of the highly-entrained IED circuitry we sought to interrogate via stimulation (Figure 1A; additional examples in Supplementary Data Sheet 1). Subjects meeting the above electrographic description were retrospectively identified via medical record review by a trained pediatric epileptologist without a priori knowledge of the analysis plan.

### 2.2 Invasive EEG acquisition

Continuous neural recordings were acquired from stereotactically-inserted depth electrodes with contacts spaced at 3.5 mm and were digitized at 512 Hz using Natus Quantum software (Middleton, WI). Electrode trajectories were determined by the clinical team and included variable coverage of mesial temporal, temporal neocortical, and extra-temporal structures (Sub01: L tempo-parietal; Sub02: diffuse L hemisphere; Sub03: bi-frontal, R parietal; Sub04: R frontal, parietal, insula). Contact locations were determined from co-registered pre-operative T1-weighted magnetic resonance imaging (MRI) and post-implantation computed tomography (CT) scans using GARDEL (GUI for Automatic Registration and Depth Electrode Localization) (Medina Villalon et al., 2018) with FreeSurfer segmentation (Dale et al., 1999). Only gray matter nodes were considered in subsequent analyses.

### 2.3 Spatiotemporal analysis of spontaneous IED bursts

For each subject, three 10-min segments of interictal activity containing abundant IEDs, minimal electrographic artifact, and separated by at least 24 h were clipped by a clinical epileptologist unaware of the study hypotheses (Figure 1). Segments were concatenated and submitted to a clinically-validated, unsupervised, automated IED detector (Brown et al., 2007), which identified IEDs based on characteristic morphologic and spectral features. A previously-described technique (Tomlinson et al., 2016; Tomlinson et al., 2019) was then used to extract IED network “bursts,” defined as the co-incident detection of IEDs across ≥15 contacts (henceforth, “nodes”) within 150 ms (Supplementary Methods). Nodes recruited in ≥30% of bursts were preserved, and bursts encompassing ≥50% of preserved nodes were analyzed further. Normalized recruitment latencies (i.e., latency (ms) from burst onset divided by total burst duration, range = 0–1) were calculated across bursts for each node and summarized using the median and interquartile range (IQR) (Figure 1C). The distribution of median recruitment latencies was used to classify nodes as upstream, intermediate, or downstream (Figure 1C, middle and right; Supplementary Methods for details). Node classifications were finalized prior to analysis of cortical stimulation to avoid bias.

### 2.4 Low-frequency stimulation for CCEPs

**FIGURE 2:**
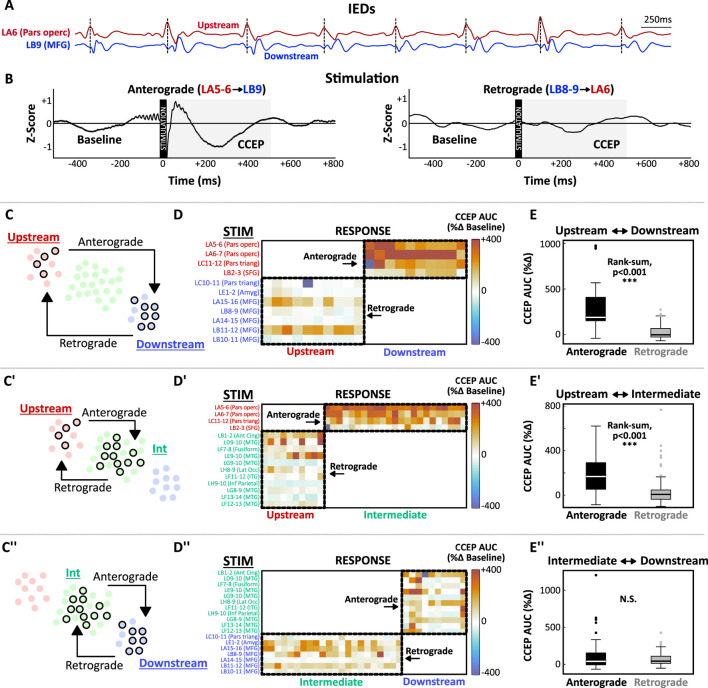
IEDs and CCEPs share common spatiotemporal constraints. **(A,B)** Illustration of the hypothesized link between IED latencies and CCEPs for representative subject (Sub02). **(A)** During IED bursts, earlier activation was consistently observed at node LA6 (pars opercularis; red, “upstream”) compared to LB9 (MFG; blue, “downstream”). **(B)** Anterograde stimulation (LA5-6→LB9) evoked a large CCEP response at LB9 (left) that was not reciprocated by retrograde stimulation (LB8-9→LA6, right). CCEP magnitude was quantified as the percent change in the area under the curve (AUC) during the post-stimulation response window (+30:500 ms). The peri-stimulus interval (–10:+20 ms) was rejected to minimize contamination from stimulation artifact. **(C)** Schematic diagram illustrating the interaction between Upstream and Downstream nodes. Stimulated nodes are depicted with black circles. Anterograde stimulation (Upstream→Downstream, four upstream stimulation nodes, 10 downstream response nodes) was compared to Retrograde stimulation (Downstream→Upstream, seven downstream stimulation nodes, 10 upstream response nodes). **(D)** Matrix encoding CCEP magnitudes for upstream-downstream node pairs. Anterograde responses (top right corner) were larger than retrograde responses (bottom left corner). **(E)** Box-plots demonstrating a significant difference between anterograde and retrograde responses (Wilcoxon rank-sum test, *p* < 0.005). These analyses were repeated for upstream-intermediate (C’–E’) and intermediate-downstream (C’’–E’’) interactions. Abbreviations: Amyg, amygdala; ant cing, anterior cingulate; AUC, area under the curve; CCEP, cortico-cortical evoked potential; IED, interictal epileptiform discharge; inf, inferior; Int, intermediate; ITG, inferior temporal gyrus; lat occ, lateral occipital; MFG, middle frontal gyrus; MTG, middle temporal gyrus; operc, opercularis; SFG, superior frontal gyrus; triang, triangularis.

Systematic low-frequency stimulation was performed by delivering 30 repeated pulses of 1 Hz biphasic stimulation to each pair of adjacent nodes (Nicolet Cortical Stimulator; pulse width: 300–500 μs; amplitude: 4–6 mA). Post-processing of raw response waveforms included peri-stimulus rejection (−10 ms to +20 ms), linear detrending, z-score normalization, and baseline subtraction (−500:−30 ms). Responses were averaged across the 30 repeated pulses to yield the grand-average CCEP waveform. The CCEP response magnitude was quantified as the percent change from baseline of the area under the curve (%Δ AUC; −500:−30 ms vs. +30:+500 ms) (Figures 2A,B). A stimulation-response matrix was used to encode the %Δ AUC for all non-artifactual CCEPs (Figure 2D).

### 2.5 Statistical analysis

We compared CCEP responses (%Δ AUC) following stimulation of upstream, intermediate, and downstream nodes at the individual-subject level using the Wilcoxon rank-sum test (α = 0.05) (Figure 2E). We predicted that CCEPs would be accentuated when stimulating upstream IED nodes (anterograde direction) and attenuated when stimulating intermediate or downstream nodes (retrograde direction). Unless otherwise noted, summary statistics are presented as mean ± standard deviation. All analyses were performed using Matlab 2024b.

## 3 Results

Diffusely-synchronous, continuous bursts of rhythmic IEDs were extracted from invasive neural recordings in four human subjects undergoing SEEG evaluation for epilepsy (Figures 1A,B). Selection of gray matter contacts participating in frequent IED bursts yielded an average of 47.0 ± 20.7 nodes for analysis (range: 27–76 nodes). Across 30 total minutes of activity per patient, IED bursts were detected at an average rate of 33.7 ± 16.4 bursts/min (range: 19.3–50.6 bursts/min). Nodes were classified as upstream, intermediate, or downstream based on their median normalized recruitment latency during IED bursts (Figure 1C).

Cortical stimulation was performed to examine the spatiotemporal relationship between spontaneous IED propagation and evoked potentials (Figure 2). The conceptual link between IED latencies and CCEPs is demonstrated in Figures 2A,B. In this example (Sub02), node LA6 (pars opercularis) consistently activated upstream of LB9 (middle frontal gyrus) during IEDs. Stimulation in the anterograde direction [LA5-6 (Upstream) → LB9 (Downstream); Figure 2B, left] elicited a robust response from LB9 which was not reciprocated by stimulation in the retrograde direction [LB8-9 (Downstream) → LA6 (Upstream); Figure 2B, right]. When all stimulation-response pairs for this patient were considered (Figures 2C–E), stimulation of upstream IED nodes (pars opercularis, pars triangularis, and superior frontal gyrus) evoked robust anterograde responses from intermediate nodes (anterior cingulate, middle temporal gyrus, inferior temporal gyrus, etc.) and downstream nodes (pars triangularis, amygdala, and middle frontal gyrus) (Figure 2D). These anterograde responses (Upstream→Intermediate and Upstream→Downstream) were significantly larger than reciprocal responses in the retrograde direction (Figure 2E, p’s < 0.001).

**FIGURE 3:**
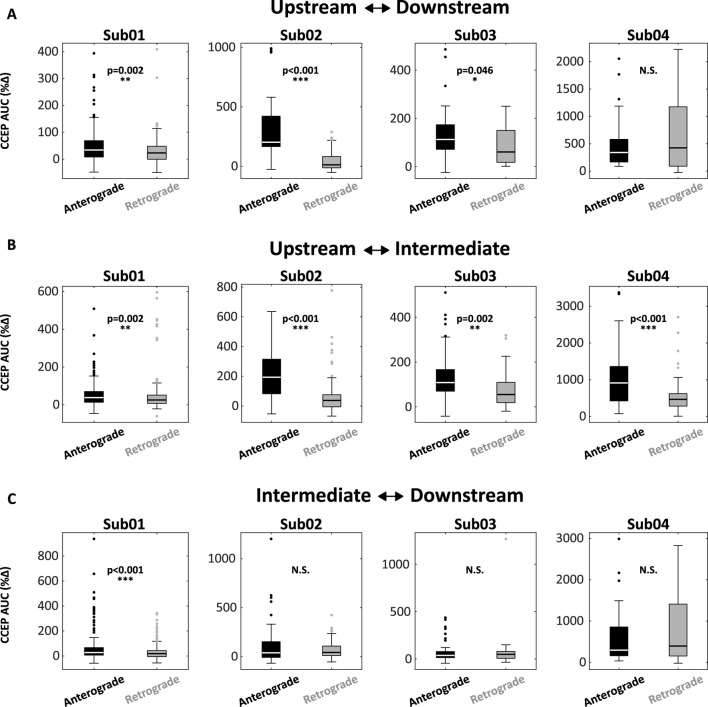
CCEPs are accentuated following anterograde stimulation and attenuated following retrograde stimulation. **(A)** Box-plots encoding CCEP responses for upstream-downstream interactions across four subjects. In 3/4 subjects (Sub01, 02, 03), anterograde responses were significantly larger than retrograde responses. **(B)** Similar differences were noted in 4/4 subjects for upstream-intermediate interactions. **(C)** Only one patient (Sub01) exhibited a difference between anterograde and retrograde responses for the intermediate-downstream interaction. Abbreviations: AUC, area under the curve; CCEP, cortico-cortical evoked potential.

Consistent findings emerged across the pilot cohort (Figure 3). In 3/4 subjects, stimulation of Upstream nodes produced a disproportionately large anterograde response in Downstream nodes (Upstream→Downstream) compared the retrograde direction (Downstream→Upstream) (Figure 3A). In all 4 subjects, anterograde responses were significantly larger than retrograde responses when Upstream-Intermediate node interactions were examined. Intriguingly, only one patient (Sub01) exhibited significant asymmetry for Intermediate-Downstream interactions, suggesting that concordance between IEDs and CCEPs is strongest for the early phase of IED recruitment.

## 4 Discussion

The epileptic brain generates a variety of spontaneous epileptiform paroxysms such as seizures, IEDs, and high-frequency oscillations (Jiruska et al., 2013; Lehnertz et al., 2009). Characterizing the networks that facilitate these phenomena, including their complex spatiotemporal organization, is critical for improving our mechanistic understanding of epilepsy and enhancing our therapeutic approach to the disorder. IEDs are a major electrographic signature of epilepsy, and researchers maintain a long-standing interest in mapping the spatiotemporal distribution of IEDs across the brain. Central to this pursuit is understanding how IEDs arise and propagate through networks of functionally-connected brain regions, and how pathways of IED transmission relate to the underlying organization of the epileptic network.

In this brief report, we provide novel experimental data demonstrating that the epileptic network imposes common spatiotemporal constraints on the spontaneous flow of IEDs and evoked cortical potentials. Although this result may seem intuitive, the potential implications are significant. First, although consistent lags between coincident IEDs have been theorized to reflect polarized pathways of functional communication between brain regions, experimental evidence supporting this assertion is lacking, especially in the context of human epilepsy. Spatiotemporal concordance between IEDs and CCEPs suggests that this polarized circuitry is a conserved feature of the epileptic network as opposed to a mere epiphenomenon or artifact of the analytic technique. Mechanistically, questions arise about how repeated IEDs may reinforce polarized network circuitry throughout the course of epileptogenesis, and whether intervening upon this process could steer the network towards less seizure-prone configurations. From a translational vantage, chronic neurostimulation has been increasingly integrated into the surgical armamentarium for refractory epilepsy, and techniques for selecting optimal stimulation targets remain uncertain. One strategy may be targeting nodes situated upstream within the spatiotemporal structure of the network such that their effective downstream “reach” is maximized. This hypothesis could be tested by comparing IED recruitment latencies at stimulation sites in patients who responded favorably versus unfavorably to responsive neurostimulation. Similarly, one could examine how surgical outcomes relate to resection of the predominant upstream IED and CCEP nodes.

Our study has several limitations. First, this proof-of-principle study included a small pilot cohort of four subjects exhibiting maximally frequent and synchronous IEDs with a consistent pattern of propagation, providing an idealized electrographic context within which to ask experimental questions about spatiotemporal network circuitry. Extending the analysis to a broader cohort with variable IED frequency and multiple independent or semi-independent IED populations is a challenging but necessary endeavor. Expanding the cohort will allow us to further investigate apparent discordances such as the unexpectedly robust activation of upstream regions following stimulation of downstream IED nodes observed in Sub04 (Figure 3A, right). This finding could reflect a truly bidirectional effective relationship or a statistical outlier within our small sample. Additionally, an expanded cohort would allow us to explore how IED and CCEP concordance varies in relation to age, seizure burden, and underlying epileptogenic substrate. Next, as with all SEEG studies, spatial sampling is limited to the clinically-determined implant targets and may under-sample the true extent of the IED network. Finally, given the high rate of IED activation, we have only analyzed three segments of EEG per patient (30-min total). The study could be expanded to include additional segments, though we note that IED recruitment patterns were very consistent across repeated discharges (Figure 1C).

In conclusion, this pilot study examined the spatiotemporal concordance between spontaneous IED activity and stimulation-evoked cortical potentials in human subjects with highly-entrained IED circuitry defined by the repetitive, sequential propagation of IEDs throughout consistent networks of functionally-connected gray matter nodes. We demonstrate that IEDs and CCEPs share common spatiotemporal constraints, such that directional asymmetries observed during IED propagation are reproduced during stimulation. Future work will extend this analysis beyond the pilot cohort and explore implications for resective epilepsy surgery and chronic neuromodulation.

## Data Availability

The raw data supporting the conclusions of this article will be made available by the authors, without undue reservation.
