# Supplementary material for: Interictal spikes and evoked cortical potentials share common spatiotemporal constraints in human epilepsy
Source: Front Netw Physiol. 2025 May 30;5:1602124. doi: 10.3389/fnetp.2025.1602124 (PMC12175433; doi:10.3389/fnetp.2025.1602124)
Supplement: Supplementary file 1 [file DataSheet1.pdf]

Supplementary Material

1 Supplementary Tables

ID	Gender	Age (y)	Etiology	IED Network Localization	Implanted contacts (N)	Gray matter nodes involved in IED bursts (N, %)	Intervention
Sub01	Female	3	FCD1a	L temporal, L parietal	144	76 (52.8)	Focal resection
Sub02	Male	3	RE	Diffuse L hemispheric	102	43 (42.2)	Hemispherotomy
Sub03	Female	6	Unknown (no resection)	R frontal, R parietal, L frontal	247	27 (10.9)	VNS
Sub04	Male	10	Unknown (no resection)	R frontal, R parietal, R insula	152	42 (27.6)	Medical therapy

Supplementary Table 1. Clinical characteristics of study subjects. *Abbreviations*: FCD, focal cortical dysplasia; IED, interictal epileptiform discharge; RE, Rasmussen's encephalitis; VNS, vagus nerve stimulation

2 Supplementary Methods

2.1. IED burst detection. A previously-described technique[1,2] was used to extract IED network "bursts," defined as the co-incident detection of IEDs across ≥15 contacts (henceforth, "nodes") within 150ms. This algorithm begins by initiating the first detected IED in the recording as the "leader" of a candidate IED burst. All IEDs occurring within 150ms of the "leader" are then appended to the candidate burst in order of increasing latency from the leader. Finally, to provide flexibility for unexpectedly long sequences extending beyond 150ms from leader, additional IEDs occurring within 5ms of the preceding IED are added to the candidate burst. If the candidate burst encompasses ≥15 unique contacts, it is preserved. After all bursts are detected from the full recording array, we next define a network of nodes consistently recruited during IED bursts. For each node in the recording array, the percentage of total bursts in which the node was recruited is calculated. Nodes recruited in ≥30% of bursts are preserved for analysis. Finally, to focus on bursts involving this consistent network of nodes, bursts encompassing ≥50% of preserved nodes are preserved for further analysis.

2.2. Node designations based on IED latencies. Normalized recruitment latencies (i.e., latency (ms) from burst onset divided by total burst duration, range = 0-1) were calculated across bursts for each node and summarized using the median and interquartile range (IQR). The distribution of median recruitment latencies was used to classify nodes as upstream, intermediate, or downstream, as described below. Node classifications were finalized prior to analysis of cortical stimulation to avoid bias.

Histograms of normalized latency values were inspected visually for each patient to aid in designation of nodes as upstream, intermediate, or downstream. We used visually-apparent peaks in the histogram distribution to determine cut-off values, which are listed for each subject below. In this example (Sub01), intuitive cut-offs can be appreciated between upstream (0-0.3), intermediate (0.3-0.7), and downstream (0.7-1.0).

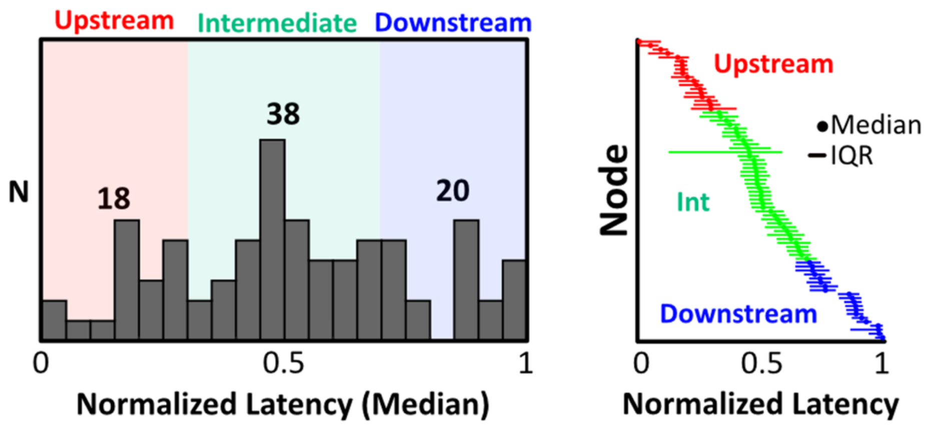

In this example (Sub04), the downstream portion of the distribution is less robust, and one could consider including only the very end of the distribution (normalized latency >0.9). However, this would only leave 2 contacts in the downstream classification, of which 1 was stimulated. To allow for more robust stimulation analysis, we extended the downstream cut-off (0.6-1.0).

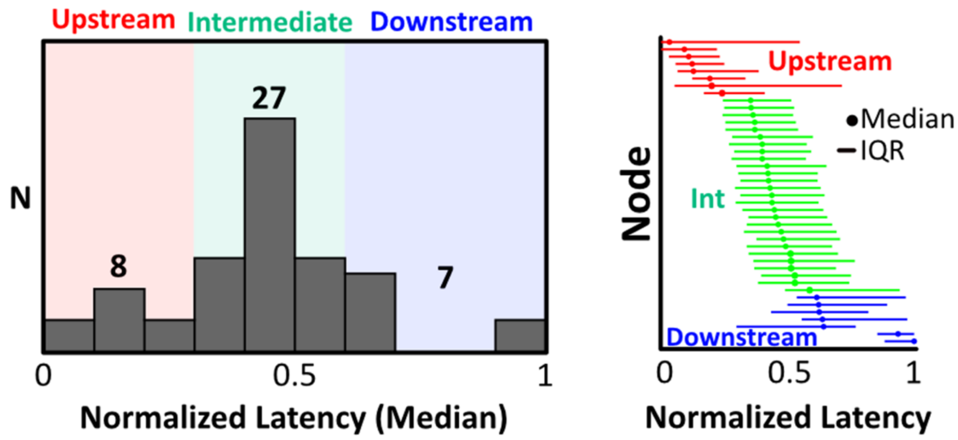

Cut-off values of normalized latency values and number of nodes (N) for each subject are listed here:

ID	Upstream (cut-off, N)	Intermediate (cut-off, N)	Downstream (cut-off, N)
Sub01	0-0.3 (18)	0.3-0.7 (38)	0.7-1.0 (20)
Sub02	0-0.3 (10)	0.3-0.7 (23)	0.7-1.0 (10)
Sub03	0-0.2 (5)	0.2-0.6 (13)	0.6-1.0 (9)
Sub04	0-0.3 (8)	0.3-0.6 (27)	0.6-1.0 (7)

3 Supplementary Figures

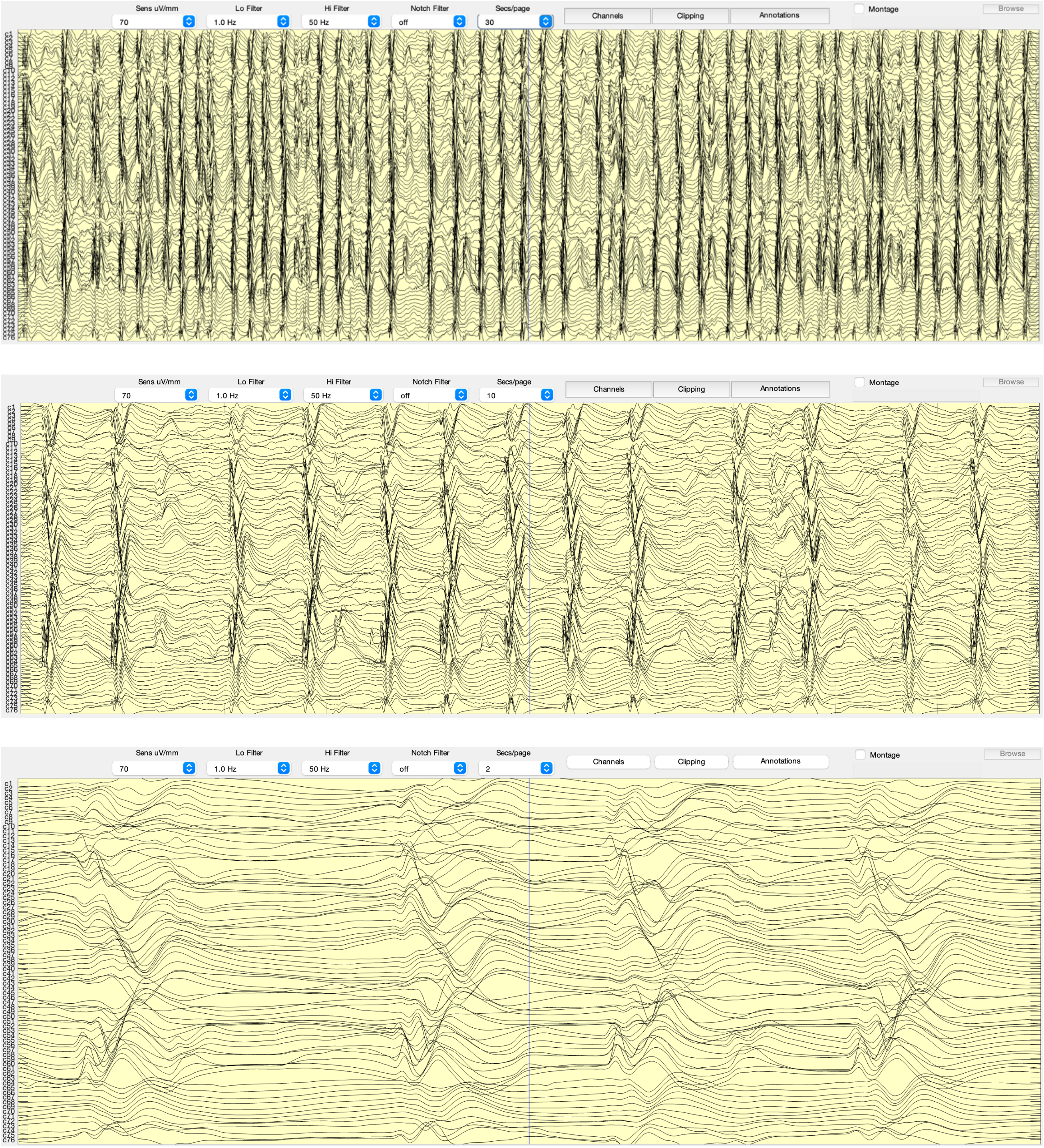

Supplementary Figure 1a. Representative IED burst activity (Sub01) demonstrating synchronous, continuous, rhythmic bursts of IEDs. Clips are 30-s (top), 10-s (middle), and 2-s (bottom) in duration. Preserved nodes (n=76) are depicted.

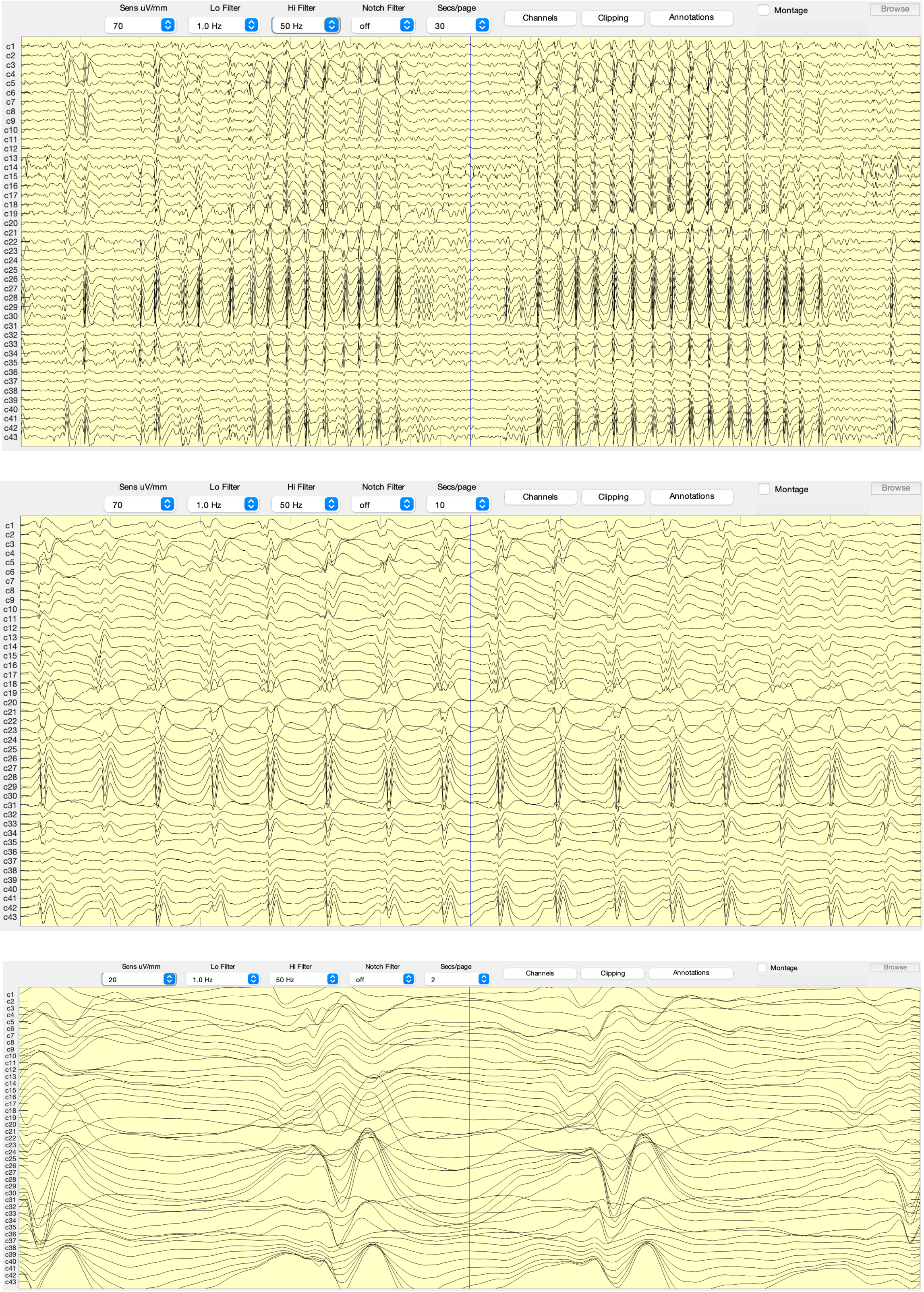

Supplementary Figure 1b. Representative IED burst activity (Sub02) demonstrating synchronous, continuous, rhythmic bursts of IEDs. Clips are 30-s (top), 10-s (middle), and 2-s (bottom) in duration. Preserved nodes (n=43) are depicted.

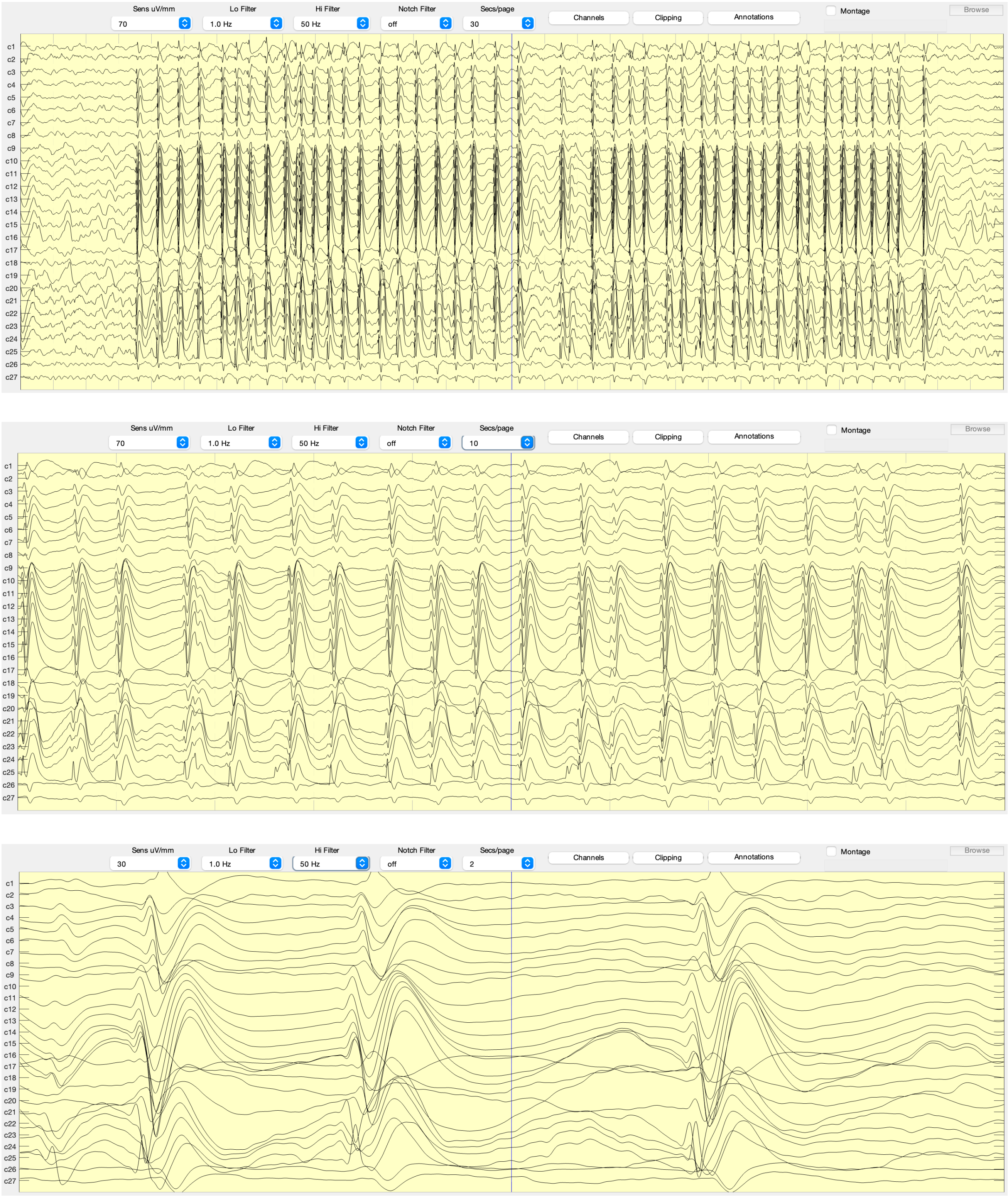

Supplementary Figure 1c. Representative IED burst activity (Sub03) demonstrating synchronous, continuous, rhythmic bursts of IEDs. Clips are 30-s (top), 10-s (middle), and 2-s (bottom) in duration. Preserved nodes (n=27) are depicted.

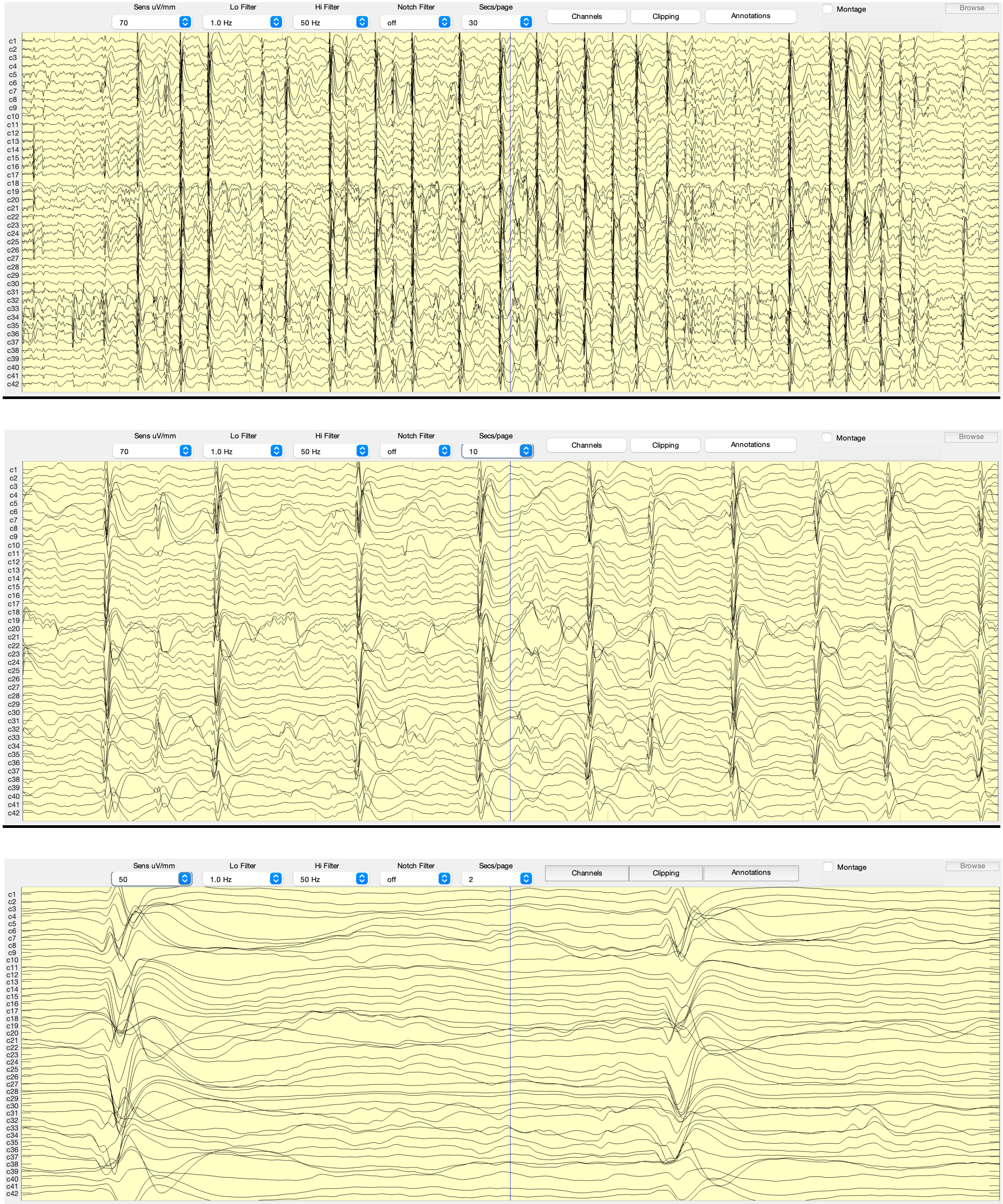

Supplementary Figure 1d. Representative IED burst activity (Sub04) demonstrating synchronous, continuous, rhythmic bursts of IEDs. Clips are 30-s (top), 10-s (middle), and 2-s (bottom) in duration. Preserved nodes (n=42) are depicted.

Supplementary References

1. Tomlinson SB, Bermudez C, Conley C, Brown MW, Porter BE, Marsh ED. Spatiotemporal Mapping of Interictal Spike Propagation: A Novel Methodology Applied to Pediatric Intracranial EEG Recordings. *Front Neurol*. 2016;7:229. doi:10.3389/fneur.2016.00229

2. Tomlinson SB, Wong JN, Conrad EC, Kennedy BC, Marsh ED. Reproducibility of interictal spike propagation in children with refractory epilepsy. *Epilepsia*. May 2019;60(5):898-910. doi:10.1111/epi.14720
